# Cerebration

**Published:** 1868-12

**Authors:** Henry M. Lilly

**Affiliations:** Fond du Lac, Wis.


CHICAGO MEDICAL EXAMINER.

N. S. DAVIS, M.D., Editor.

VOL. IX. DECEMBER, 1868. NO. 12.

### Original Contributions.

ARTICLE XLVIII.

### "CEREBRATION."

By HENRY M. LILLY, M.D., of Fond du Lac, Wis., Nov. 4th, 1868.

When we propose to use a scientific term in the discussion of a subject requiring close reasoning, it is a good thing to settle beforehand exactly what we mean by the term; and also whether the meaning we attach to the term is merely an uncertain hypothesis, or actually a proven fact. To call the function of the cerebrum "*cerebration*," and then to say that cerebration is thinking, and that thinking is just as much a product of the cerebrum as bile is of the liver, is a mode of shifting terms about, with the interpolation of an assertion or two, that leaves us no wiser than we were at the outset.

No physiologist doubts that the healthy action of the cerebrum (whatever that action may be) is an indispensable condition to the healthy action of the mind in a living man. But wherein are we enlightened, by calling this action of the hemispherical ganglion "cerebration"? Certainly there is no objection to the use of the term, if we limit its meaning so that it shall cover nothing except what we know of cerebral function. But when a writer in the August number of the Medical Examiner tells us that "ratiocination and ideation are but the result of the moulting and regeneration of cerebral cells," he is covering by the term "cerebration" not only all that we know of cerebral function, but a good deal besides.

Physiologists believe that our optic ganglia (the tubercula quadrigemina) are in some way indispensably connected with the sensation of sight. The image of a man walking upon the sidewalk is formed upon our retina; and we know how this is done. We know, also, the simple fact that through some agency of the optic nerve this image and the optic ganglia are brought somehow into connection; but we *don't* know how this is done. We know, moreover, that through the help of our optic ganglia we obtain the *sensation* of the image upon the retina; but we are utterly ignorant as to how *this* is done. To say that the sensation of sight is but the result of the moulting and regeneration of the cells of the optic ganglia, would not bring us a single step nearer to a satisfactory solution. It is, of course, difficult to refute such assertions, because we are all equally in the dark. If we knew how our optic ganglia helped us to see, and how our cerebrum helped us to think, then all such assertions and hypotheses would be either proven true or proven false, without any farther ado. But in the present state of our knowledge they only serve to mystify still more a darkness already sufficiently mysterious.

If we seek only to find how the impressions made upon our senses are conveyed to the ganglia of the senses by the nerves, we find ourselves involved in Egyptian darkness. If we seek next to ascertain how those impressions thus transmitted to these ganglia become there converted into sensations, lo! the darkness is more than doubled upon us. Lastly, if we desire to know how with our cerebrum we take cognizance of these sensations formed by the help of the sensory ganglia, it is as if the sun and all the stars were blotted out from the heavens. Who now will believe us if we call to our fellows from this outer darkness, "Ah, we see! mind results from organization; it is made by the cerebrum just as bile is made by the liver?" If any portion of the world believes the statement, it will believe simply because it takes a notion to, and not because it sees any light ahead.

Men believe that matter and mind are in their essences two entirely different things, not because they know anything about the essence of either mind or matter, but because they perceive easily that the properties of the two are totally unlike. They see that hope, fear, and love, memory, self-consciousness and reasoning, cannot in any way be compared with the properties of a certain number of ounces of bile, nor with the properties of the electric force. Hence, they will continue to believe that mind is something besides a secretion or a force produced by matter, until they are convinced to the contrary by a satisfactory demonstration.

Every man carries with him a certain self-consciousness that his individual self is something quite different from his body, or any part of his body. He does not believe that his body and its products are all there is of him, or even constitute his personality. He looks upon his hands as not himself, but certain instruments of his. He looks upon his liver and his brain in the same way. None of us can explain this self-consciousness. None of us can prove from the study of organization whether we are, or are not, woefully deceived by it. But the fact of its existence is incontrovertible; and until this notion is knocked out of us by the stern logic of scientific demonstration, we must accept it as a probably correct notion.

Very many of the inferior animals possess a cerebrum, and exhibit more or less of mental traits differing in degree only from those exhibited by man. The cerebrum of the horse is doubtless similar in function to that of man. If the cerebrum of man is an instrument of the mind, then also the cerebrum of the horse is an instrument of mind. If memory is an attribute of mind, then a horse has a mind. If by the term mind we mean the "soul," then a horse has a "soul." Whether we study this animal anatomically or psychologically, we are led to the same conclusion. But this does not prove that mind is the result of organization. It simply transfers the argument to other grounds, and leaves the whole question "*in statu quo.*"

Because as anatomists and physiologists we have no knowledge of mind apart from a cerebrum, it follows logically that

*as anatomists and physiologists* we cannot trace the existence of mind beyond the death of the cerebrum. Are we, therefore, justified in drawing the conclusion that "the idea that man may retain the power of thought after the destruction of the brain is too absurd to bear the test of careful scrutiny"? This question is taken by the hand of death entirely from the domain of the physiologist as such. It becomes now simply a question of the power and purposes of the Creator. A Power sufficient to create a mind is certainly sufficient to continue its existence after the destruction of the brain, if it chooses to do so. If it can create the brute mind with the commencement of organic life, it can destroy the brute mind at the termination of organic life, if it chooses to. If it can create the mind or soul of man with the organic life of the body, it is certainly capable of either destroying it with the body, or perpetuating it after the death of the body, at its option.

If we believe in the existence of a God, then we believe in the existence of a mind, concerning which it would be a logical absurdity to say that it was itself only the product of organization. But if the mental attributes of the Deity can exist without a cerebrum, then it follows that the mental attributes of man are not necessarily, nor even probably, the products of his cerebrum.

On the other hand, if "ideation and ratiocination are but the results of the moulting and regeneration of cerebral cells,"—if thought is but the bursting of a cerebral cell,—if, in short, *mind* is only the result of organization, *then* it follows that there is no God in the universe. Everything in the universe, with all the beautiful and transcendently perfect adaptations that we see in Nature, are the result of *what?*—shall we say "of organization?" But whence came the organization? No man can deny or disbelieve that the organization, for example, of the cerebrum and all other portions of the body of man display *intelligence*,—display intelligent *forethought* and wisdom. Then intelligence is *antecedent* to organization, and is not necessarily a product of it. But if intelligence is antecedent to organization in *any* known case, by what authority do we claim it to be the product of organization in some other case? Our logic becomes at once as philosophical as the performance of a playful dog, running around in an animated pursuit of the end of his tail. The horse does not pull the cart, but the cart propels the horse;—that's the way the whole concern comes to *move along*,—says our philosopher. But if you ask him how the cart comes to push the horse, he says,—Oh, that is the result of organization. Now, if you ask him how the organization came to be so well adapted to move the cart that pushes the horse, he replies by asking you to explain to him something else which you don't understand. Now, how much wiser, or better, or more probably truthful, is this theory than that of the little child who will tell you that God made the horse and the horse pulls the cart, and that is all that he knows about it? The logical result of all this absurd theory of cerebration is, that there is no God at all in the universe,—that our personal identity is nothing but a product of our cerebrum, so far as we know anything about it,—that there is no mind in us except what results from "cerebration," just as there is no milk possible except what results from lactation. If these be the "lookout mountains" of science upon which we are invited to "plant our feet firmly," verily, our battles on the subject will not be fought a great way above the clouds. The theory of "unconscious cerebration" is certainly not a "*startling* theory." What is there "startling" in the theory that our cerebrum may perform its functions without our being conscious of the action? Are we *ever* conscious of the action either of the cerebrum or of the liver? That the *mind* may act unconsciously, is a curious and interesting fact, and one worthy of investigation.

Our writer on "Unconscious Cerebration" is entitled to our thanks for the very interesting facts he brings forward on this subject. But this unconscious action of the mind does not prove that "mind-force is correlative nerve-force," nor that mind is a product of the cerebrum. If the mind *can* act, and *does* act, unconsciously,—as we admit,—and if, in the living man, the functional activity of the cerebrum is an indispensable condition of mental action, as we all admit, then it follows necessarily that "*cerebration*" may take place unconsciously. But whatever may be said either in affirmation or negation upon this question of unconscious action does not in the least affect the question as to what cerebration is. The question of unconsciousness does not affect the question of the nature of the action itself. After we have said all that we wish upon the former question, the latter will still roll back upon us with neither more nor less of its pristine force.

